# Implication du pollen de canne à sucre dans les manifestations de la rhinite allergique: étude cas témoins

**DOI:** 10.11604/pamj.2022.41.133.27897

**Published:** 2022-02-16

**Authors:** Patrick Maholisoa Randrianandraina, Corinne Eulalie Solo, Ravaka Hariniaina Andriambelo, Mamy Jean Jacques Razafimahatratra, Heritsilavo Eloi Ramilison, Miora Christine Mamiharilala, Andriarimanana Hery Nirina Rakotoarisoa

**Affiliations:** 1 Service d’Oto-Rhino-Laryngologie du Centre Hospitalier Universitaire Professeur Zafisaona Gabriel, Mahajanga 401, Madagascar,; 2 Service de Chirurgie Générale, Centre Hospitalier Universitaire Tanambao, Antsiranana 201, Madagascar,; 3 Service d´Ophtalmologie du Centre Hospitalier Universitaire Anosiala, Antananarivo 101, Madagascar; 4 Institut National de Santé Publique et Communautaire, Faculté de médecine d´Antananarivo, Antananarivo 101, Madagascar,; 5 Service d´Oto-Rhino-Laryngologie du Centre Hospitalier Universitaire Place Kabary, Antsiranana 201, Madagascar,; 6 Faculté de médecine d´Antsiranana, Antsiranana 201, Madagascar

**Keywords:** Allergène, pollinose, allergie respiratoire, canne à sucre, Allergen, pollinosis, respiratory allergy, sugarcane

## Abstract

L´identification des allergènes est essentielle dans la prise en charge de la rhinite allergique. La canne à sucre produit un pollen anémophile. L´objectif de notre étude était de déterminer la place du pollen de canne à sucre dans la survenue de la rhinite allergique. Il s´agit d´une étude analytique type cas-témoins, menée de juillet 2017 à juin 2018, chez des patients habitants une commune rurale malgache dans laquelle la culture et le traitement de la canne à sucre constituent les principales occupations. Elle incluait 182 patients (91 cas et 91 témoins). Les facteurs associés à la survenue des signes de rhinite allergique étaient la distance de moins de 500 mètres entre les domiciles et les champs de canne à sucre (OR = 1,50), la profession exercée en contact direct avec la canne à sucre (OR=116) et la présence d´antécédent familial de rhinite allergique (OR=13,67). Par ailleurs, l´exposition aux rafales (OR=0,84) et la profession exercée en plein air (OR=0,92) étaient des facteurs protecteurs. L´exposition des patients au pollen de canne à sucre est associée aux manifestations cliniques de la rhinite allergique et confirme la place que tient cet allergène dans la survenue de ladite pathologie. Des mesures d´éviction et d´hygiènes constituent par conséquents la base du traitement.

## Abstract (en)

The identification of allergens is essential in the management of allergic rhinitis. Sugarcane produces anemophilic pollen. The purpose of our study is to assess the role of sugarcane pollen in the occurrence of allergic rhinitis. We conducted a case-control analytical study of patients living in a Malagasy rural commune in which sugarcane cultivation and processing are important sources of employment, from July 2017 to June 2018. We enrolled 182 patients (91 cases and 91 controls). Factors associated with the occurrence of symptoms of allergic rhinitis were: a distance of less than 500 meters between homes and sugarcane fields (OR = 1.50), being a sugarcane worker (OR=1.16) and having a family history of allergic rhinitis (OR=13.67). In addition, exposure to wind gusts (OR=0.84) and outdoor occupation (OR=0.92) were protective factors. Exposure of patients to sugarcane pollen is associated with clinical manifestations of allergic rhinitis and confirms the role of this allergen in the occurrence of the disease. Avoidance and hygiene measures are the basis of treatment.

## Introduction

**Contexte:** la rhinite allergique correspond à un ensemble de manifestations nasales résultant d´une réaction immunologique IGE-dépendante, consécutif à une exposition de la muqueuse nasale à un allergène. C´est une pathologie fréquente en pratique médicale, et dont la prévalence dans la population générale varie selon les régions, soit de 5 à 50% en Europe [1] et de 10 à 35,7% en Afrique [2, 3]. Cette prévalence serait actuellement en perpétuelle augmentation et constitue un problème de santé publique [3]. En effet, les manifestations cliniques de la rhinite allergique peuvent d´une part être responsables d´une altération majeure de la qualité de vie, du rendement professionnel et scolaire, et d´autre part être précurseurs d´asthme engageant le pronostic vital [1, 4]. Étant multifactorielle, la prise en charge dépend surtout de l´éviction des allergènes et de la maîtrise de l´environnement [5]. Les formes de rhinite allergique présentées par les patients vus en consultation sont prises en charge sans que nous disposions d´une orientation étiologique claire, car les travaux publiés à ce sujet sont insuffisants. Étant donné que l´exposition au pollen de canne à sucre concerne plusieurs milliers de personnes dans différentes régions de Madagascar, l´objectif de notre étude était de déterminer la place du pollen de canne à sucre dans la survenue des manifestations cliniques de la rhinite allergique.

## Méthodes

**Conception de l´étude:** il s´agit d´une étude analytique type cas-témoins.

**Cadre de l´étude:** réalisée à Ankaratra ou «Sirama», une commune rurale du district d´Ambilobe, dans la Région DIANA, Madagascar, l´étude s´étendait sur une période de 11 mois, allant de juillet 2017 à juin 2018. L´économie de ladite commune tourne principalement autour de la culture, du traitement et de la transformation de la canne à sucre, dont la période de floraison se situe entre les mois d´avril et d´août. Le site d´étude est par ailleurs sous l´effet de l´Alizé ou «Varatraza», constitué de vents forts, soufflant d´avril à octobre.

**Participants:** les «cas» regroupaient les personnes habitants la commune de Sirama et ses environs, présentant des signes cliniques de rhinite allergique (association variable de rhinorrhée claire, obstruction nasale, éternuements, prurit nasal ou vélo-pharyngé avec ou sans signes ophtalmologiques) lors de la période de floraison et d´exploitation des cannes à sucres. Les «témoins» étaient les personnes habitants dans la commune de Sirama et ses environs, ne présentant pas de signes de rhinite allergique. Les cas et les témoins étaient recrutés pendant la même période. Les patients présentant des tumeurs endonasales ou de rhinite inflammatoire non allergique ou qui souhaitaient ne pas faire partie de l´étude n´étaient pas inclus. La sélection des «témoins» était faite par appariement avec les «cas» selon l´âge, le genre, le domicile des patients, avec un ratio cas/témoins de 1/1.

**Variables:** en plus des variables épidémiologiques, les variables étudiés chez les cas et les témoins étaient les périodes de manifestations des signes cliniques pendant l´année, les antécédents personnels et familiaux d´allergie, les facteurs d´exposition au pollen de canne à sucre caractérisés par la proximité des domiciles avec les champs, le niveau d´exposition au pollen de canne à sucre selon le type du lieu d´exercice du travail, la durée journalière de travail et l´exposition aux rafales. Les facteurs environnementaux des cas et des témoins étaient la durée du temps passée au domicile par jour, le niveau d´insolation des habitats, la fréquence quotidienne de nettoyage des domiciles, l´existence d´exposition aux odeurs fortes et aux animaux domestiques.

**Sources de données:** les données étaient collectées au cours de consultations de masses sur bases d´appels médias, à partir de fiches d´enquête anonymes. Les patients profitaient au préalable de consentement éclairé, aboutissant à un accord écrit, tenus dans un lieu assurant la protection de leur identité. Un interrogatoire individuel des cas, orienté par un questionnaire orienté et préétabli a permis d´obtenir les variables. Il en était de même pour les témoins.

**Taille de l´étude:** le calcul de la taille de l´échantillon ainsi que le traitement des données étaient effectués à l´aide du logiciel Epi info version 7.1.3.

**Méthodes statistiques:** l´Odds Ratio (OR) avec un Intervalle de Confiance (IC) à 95% était la mesure d´association qui permettait d´évaluer la relation entre la rhinite allergique et les facteurs associés. Le test de chi carré de Pearson était utilisé pour la comparaison des proportions. Le seuil de significativité statistique était fixé à ≤0,05.

## Résultats

**Participants:** sur 205 patients éligibles, la participation à l´enquête a été refusée par 4 cas et 19 témoins. Nous avons ainsi retenu 182 patients dont 91 cas et 91 témoins.

**Données descriptives:** l´âge moyen (± écart type) des «cas» était de 30,8 ans (± 15,97), avec un sex-ratio de 0,68 (soit 37 hommes pour 54 femmes).

**Figure 1:**
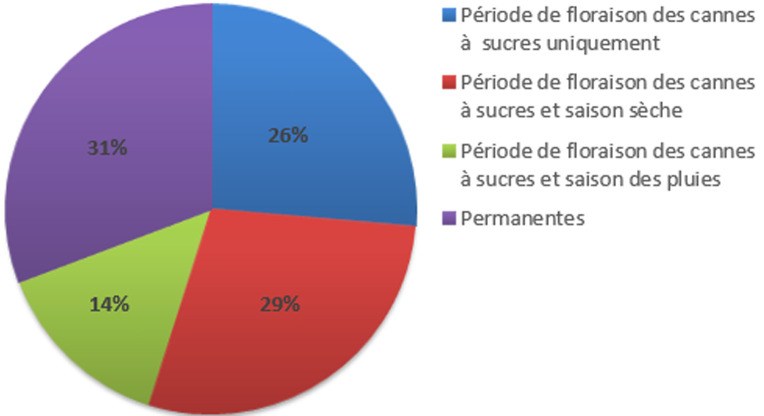
répartition des patients selon les périodes de manifestations des signes cliniques dans l’année

**Données obtenues:** tous les patients «cas» présentaient des manifestations de la rhinite allergique pendant la période de floraison de la canne à sucre (avril à août). La répartition des patients selon les périodes de manifestations des signes de rhinite allergiques durant l´année d´étude est rapportée sur la Figure 1.

**Principaux résultats:** les relations entre des facteurs d´exposition au pollen de canne à sucre chez les patients sont rapportées dans le Tableau 1. Le niveau d´exposition fort correspond à une profession exercée en contact direct avec la canne à sucre. Le niveau moyen est une profession exercée en plein air sans contact direct avec la canne à sucre. Le niveau faible est une profession exercée dans des locaux à l´abri de la canne à sucre. Les antécédents allergiques des patients sont rapportés dans le Tableau 2. Les caractéristiques de l´environnement et les facteurs environnementaux des patients sont rapportées dans le Tableau 3.

**Tableau 1:** facteurs d’exposition au pollen de canne à sucre

Facteurs	CasN= 91 (100%)	Témoins N= 91 (100%)	OR [ICà95%]
Distance Domicile-Champs de canne à sucre			
≤ à 500 mètres	43 (47,3%)	34 (37,4%)	1,50 [0,83-2,71]
> à 500 mètres	48 (52,7%)	57 (62,6%)	
Niveau d'exposition selon la profession			
Forte	14 (15,4%)	12(13,2%)	1,16 [0,49-2,75]
Moyen	23 (25,3%)	25 (27,5%)	0,92 [0,46-1,81]
Faible	54 (59,3%)	54 (59,3%)	
Durée journalière de travail			
≤ à 8 heures	75 (82,4%)	71 (78%)	1,32 [0,63-2,74]
> à 8 heures	16 (17,6%)	20 (22%)	
Exposition intermittente aux rafales de vents			
Exposés	84 (92,3%)	85 (93,4%)	0,84 [0,27-2,62]
Non exposés	7 (7,7%)	6 (6,6%)	

**Tableau 2:** antécédents allergique de patients

Antécédents	CasN= 91 (100%)	Témoins N= 91 (100%)	OR [IC-95%]
Antécédents personnels d'asthme			
Avec	4(4,4)	1(1,1)	4,13 [1,45-37,76]
Sans	87(95,6)	90(98,9)	
Antécédent Familial d'asthme			
Avec	9(9,9)	1(1,1)	9,87 [1,22-79,66]
Sans	82(90,1)	90(98,9)	
Antécédent familial de Rhinite allergique			
Avec	12(13,2)	1(1,1)	13,67 [1,73-107,50]
Sans	79(86,8)	90(98,9)	

**Tableau 3:** facteurs environnementaux des patients

Facteurs	CasN= 91 (100%)	Témoins N= 91 (100%)	OR [IC-95%]
Temps quotidien passé au domicile			
Inférieur ou égale à 16 heures	76(83,51)	64(70,32)	2,13 [1,04-4,36]
Supérieur à 16 heures	15(16,48)	27(29,67)	
Niveau d´insolation des habitats			
Faible	27(29,7)	23(25,3)	1,24 [0.64-2,39]
Bon	64(70,3)	68(74,7)	
Fréquence quotidienne de balayage des habitats			
Inférieur ou égale à 1	69(75,82)	68(74,73)	1,06 [0,54-2,08]
Supérieur	22(24,18)	23(25,27)	
Exposition aux odeurs fortes			
Exposés	43(47,3)	38(41,8)	1,24 [0,69-2,24]
Non Exposés	48(52,7)	53(58,2)	
Existence d´animaux domestiques à domicile			
Oui	20(21,98)	22(24,18)	1,13 [0,56-2,25]
Non	71 (78,02)	69 (75,82)	

## Discussion

**Résultats clés:** dans cette étude menée dans une ville sucrière, des facteurs climatiques et environnementaux comme la période de floraison des cannes à sucres, un confinement à domicile de moins de 16 heures (OR=2,13), la proximité du domicile avec les champs de canne à sucre (OR=1,50), la proximité des travailleurs avec les champs de canne à sucre (OR=1,16) augmentaient le risque de présenter des manifestations de rhinite allergique. Par ailleurs, l´exercice du travail en plein air (OR= 0,92) et l´exposition des patients aux rafales intermittents (OR=0,84) étaient des facteurs protecteurs de la survenue des manifestations de la rhinite allergique.

**Limites:** les limites de cette étude étaient constituées d´une part par le mode de recueil des données au moyen d´un questionnaire. Cette méthode exposerait à des biais de mémoire, quant à la survenue de la symptomatologie dans le temps. Pour limiter ce biais, le questionnaire a été rédigé dans l´objectif de limiter ces oublis. D´autre part, l´absence de test allergologique limitait le recrutement des cas et des témoins permettant de confirmer ou éliminer l´implication des différents allergènes dans la survenue de la rhinite allergique. Les patients recrutés étaient alors sélectionnés uniquement s´ils présentaient des signes de rhinites allergiques au cours des périodes de floraisons de cannes à sucres.

**Interprétation:** la survenue de la rhinite allergique pourrait être associée à un facteur environnemental propre à chaque région. La canne à sucre est une graminée des pays tropicaux, produisant des pollens anémophiles, à l´origine de pollinoses [6]. La rhinite aux pollens de graminées correspond aux manifestations cliniques de rhinite allergique pendant la pollinisation des espèces [7]. La plupart des pollinoses surviennent au cours des saisons humides et de fortes pluies, au cours desquelles les allergènes anémophiles trouvent leurs conditions physiologiques de sensibilisation [6, 8]. En effet, étant hydrosolubles, les pollens hydratés produisent des lysozymes dont les protéases, qui peuvent faciliter le transport des protéines responsables de la réactivité de la muqueuse nasale [9]. Notre région d´étude située dans le nord de Madagascar, avec un climat tropical chaud et humide, est recouverte de plantes de canne à sucre sur près de 90% du territoire. Les manifestations cliniques de la rhinite allergique chez certains patients à Sirama ont été rapportées pendant les autres saisons de l´année, en plus des périodes de floraisons des cannes à sucres. La particularité du climat chaud et humide en zone tropicale favoriserait une concentration plus élevée des aéroallergènes dans l´air par rapport aux zones tempérées [10]. Cette aérobiose particulière en zone tropicale serait responsable d´une plus forte pollinose. Cependant, peu d´études documentées ont été effectuées sur la pollinose en milieu tropicale.

La promiscuité, le faible niveau d´insolation, l´humidité, l´insuffisance de nettoyage des domiciles et la présence d´animaux domestiques [11] sont des facteurs ménagers qui privilégient le développement d´acariens. Ce sont les principaux allergènes pourvoyeurs d´allergie respiratoire [12, 13]. Il en est de même pour l´accumulation des poussières de maison qui contiennent des allergènes respiratoires dont les moisissures et les pollens [3, 14]. Par ailleurs, le pollen de canne à sucre ne se trouve pas dans la batterie de test des allergènes couramment utilisée pour l´exploration de l´hypersensibilité nasale et des allergies en général alors que plusieurs allergènes peuvent être impliqués dans la survenue des manifestations de la rhinite allergique [6, 8]. Une potentialisation de l´effet allergique de l´association des aéroallergènes domestiques et du pollen de canne à sucre devrait ainsi être recherchée en cas d´hypersensibilité nasale [15].

L´exposition intense et prolongée aux allergènes entraine une inflammation chronique de la muqueuse nasale a minima. Cette inflammation chronique est responsable d´une perpétuation des symptômes de la rhinite allergique [7, 16]. Cependant, d´après nos résultats, l´exposition de moins de 8 heures au pollen de canne à sucre, augmentait le risque de développer des manifestations de rhinite allergique. Cette divergence pourrait être due à des caractéristiques spécifiques du pollen de canne à sucre, hypothèse qui nécessiterait une vérification par des études immunologiques poussées.

Les rafales en régions tropicales ont été rapportés comme étant des facteurs protecteurs contre la survenue de rhinite allergique [11]. En région rurale comme à Sirama, le risque d´être en contact avec les polluants atmosphériques est moindre en région rurale, évitant ainsi l´apparition de rhinite allergique [17]. C´est un avantage lié au climat de la région d´étude, car l´alizé qui souffle pendant la période de floraison des cannes à sucre, entrainerait un brassage de l´air, lui débarrassant de toute particule en suspension dont les allergènes [18].

**Généralisabilité:** l´introduction du pollen de canne à sucre dans la batterie de test des allergènes couramment utilisée pour l´exploration de l´hypersensibilité nasale et des allergies en général pourrait conforter l´hypothèse de son implication dans la survenue de rhinite allergique. Une étude de l´aérobiologie dans notre région et en zone tropicale permettrait de quantifier et de prédire la participation quotidienne éventuelle des aéroallergènes dans la survenue des manifestations de la rhinite allergique [19, 20]. Cette aérobiologie permettra de prévenir la survenue des symptômes y afférent.

Concernant les mesures de préventions, l´ensoleillement des habitats, l´éviction des poussières de maisons et des animaux domestiques sont des mesures connues pour la prise en charge des rhinites allergiques [5], quelles que soient les étiologies, avec ou sans implication du pollen de canne à sucre. Par ailleurs, la rhinite allergique au pollen de canne à sucre pourrait être considérée comme une maladie professionnelle, justifiant des mesures de protections adéquates. Vu le pouvoir protecteur des rafales dans la survenue des manifestations de la rhinite allergique, il est judicieux de recommander aux professionnels de la canne à sucre de travailler dans un espace suffisamment aéré pour prévenir l´exposition au pollen de canne à sucre.

## Conclusion

La présente étude permettait de constater que la survenue des manifestations cliniques de rhinite allergique coïncidait avec la période de floraison de la canne à sucre. La proximité de l´exposition des patients aux champs de cannes à sucres a été démontrée comme étant associé à la survenue des manifestations cliniques de la rhinite allergique et confirme la place que tient cet allergène dans les manifestations de ladite pathologie. Cependant, l´implication d´autres facteurs constitutionnels et domestiques requiert une analyse multivariée. Certains facteurs environnementaux locaux semblent protéger contre la survenue des manifestations cliniques de rhinite allergique. Des recherches fondamentales en immunologie sur l´exposition au pollen de canne à sucre permettront de démontrer le mécanisme de cette allergie et de produire des tests allergologiques incluant le pollen de canne à sucre. Ces données permettront de faciliter le diagnostic et de développer l´immunothérapie contre le pollen de canne à sucre.

### Etat des connaissances sur le sujet

- Les pollens de graminées dont le pollen de canne à sucre réveillent les signes cliniques de rhinite allergique lors de la pollinisation, mais peu de travaux sur le pollen de canne à sucre y ont apportés des preuves suffisantes;
- L´exposition prolongée aux allergènes est responsable d´une perpétuation des symptômes de la rhinite allergique.

### Contribution de notre étude à la connaissance

- Ce travail apporte des preuves de l´implication du pollen de canne à sucre dans la survenue des manifestations de la rhinite allergique à travers une étude cas-témoins;
- L´exposition de moins de 8 heures au pollen de canne à sucre, augmente le risque de développer des manifestations chroniques de rhinite allergique. Cette moindre durée d´apparition des symptômes associée aux facteurs climatiques tropicaux pourrait être en relation avec des caractéristiques spécifiques de du pollen de canne à sucre.

## Remerciements

Nous remercions tous ceux qui, de près ou de loin, ont participé à l´élaboration de ce travail.
